# Correction

**DOI:** 10.1080/07853890.2023.2245611

**Published:** 2023-09-06

**Article title:** Association of the endothelial nitric oxide synthase (eNOS) 4a/b polymorphism with the risk of incident diabetic retinopathy in patients with type 2 diabetes mellitus: a systematic review and updated meta-analysis

**Authors:** Shi Y., Fan, X., Zhang, K., & Ma, Y.

**Journal:** *Annals of Medicine*

**Bibliometrics:** Volume 55, Number 01, 2226908

**DOI:** http://dx.doi.org/10.1080/07853890.2023.2226908

When the above article was first published, some of the overall case/control numbers in Table 3 and Table 4 were incorrect due to mistakes made when summing the case/control numbers from the original studies. This error only affected the case/control summary information presented in Table 3 and Table 4.

The correct tables can be found below:

**Table 3.** Results of overall population and ethnicity-based subgroup analysis of eNOS (4b/4a) polymorphism and DR risk in T2DM patients.

Variables		*N*	References	Case/Control	Genetic model	Fixed-effects model		Random-effects model		Heterogeneity		Egger’s Test (*p*)
						OR (95%CI)	*p* value	OR (95%CI)	*p* value	P_H_	*I*^2^ (%)	
Overall					Allelic model (a versus b)							
	Total	19	[4,8,10,11,15,16,18,19,22–24,28,36–42]	3859/3979		0.973(0.897–1.056)	0.517	0.995(0.849–1.167)	0.953	0.000	67.6%	0.750
	HWE	16	[4,8,10,11,15,16,18,22,28,36–42]	3458/3602		0.952(0.874–1.038)	0.265	0.972(0.812–1.163)	0.754	0.000	70.9%	0.802
Ethnicity												
Asians	Total	11	[4,8,10,11,16,19,23,28,40–42]	2093/1977		0.925(0.826–1.035)	0.173	0.934(0.766–1.138)	0.496	0.025	51.2%	–
	HWE	9	[4,8,10,11,16,28,40–42]	1819/1710		0.9067(0.804–1.022)	0.108	0.911(0.722–1.151)	0.436	0.018	56.7%	–
Caucasians	Total/HWE	5	[15,22,36,37,39]	1188/1102		1.284(1.094–1.506)	0.002	**1.273(1.006–1.610)**	**0.045**	0.084	51.4%	–
Tunisians	Total	2	[24,38]	510/599		0.841(0.691–1.024)	0.085	0.958(0.523–1.755)	0.891	0.008	85.9%	–
Han Chinese	Total	5	[11,23,40–42]	637/752		1.130(0.888–1.438)	0.322	1.145(0.704–1.862)	0.585	0.005	73.3%	–
Overall					Recessive model (4a4b + 4b4b versus 4a4a)							
	Total	19	[4,8,10,11,15,16,18,19,22–24,28,36–42]	3859/3979		0.990(0.809–1.210)	0.918	0.969(0.738–1.271)	0.818	0.198	21.1%	0.969
	HWE	16	[4,8,10,11,15,16,18,22,28,36–42]	3458/3602		0.981(0.791–0.217)	0.863	0.944(0.712–1.253)	0.692	0.259	17.0%	0.767
Ethnicity												
Asians	Total	11	[4,8,10,11,16,19,23,28,40–42]	2093/1977		1.056(0.795–1.404)	0.706	1.079(0.804–1.446)	0.613	0.524	0.0%	–
	HWE	9	[4,8,10,11,16,28,40–42]	1819/1710		1.107(0.820–1.494)	0.507	1.098(0.809–1.490)	0.550	0.761	0.0%	–
Caucasians	Total/HWE	5	[15,22,36,37,39]	1188/1102		**0.575(0.371–0.892)**	**0.014**	0.568(0.363–0.889)	0.013	0.388	3.3%	–
Tunisians	Total	2	[24,38]	510/599		1.470(0.930–2.325)	0.099	1.468(0.928–2.323)	0.101	0.764	0.0%	–
Han Chinese	Total	5	[11,23,40–42]	637/752		0.710(0.323–1.562)	0.394	0.694(0.302–1.594)	0.389	0.697	0.0%	–
Overall					Dominant model (4a4a + 4a4b versus 4b4b)							
	Total	19	[4,8,10,11,15,16,18,19,22–24,28,36–42]	3859/3979		0.960(0.870–1.059)	0.410	0.988(0.818–1.192)	0.896	0.000	67.6%	0.619
	HWE	16	[4,8,10,11,15,16,18,22,28,36–42]	3458/3602		0.928(0.836–1.029)	0.156	0.946(0.772–1.160)	0.597	0.000	68.7%	0.767
Ethnicity												
Asians	Total	11	[4,8,10,11,16,19,23,28,40–42]	2093/1977		0.903(0.788–1.036)	0.145	0.925(0.749–1.142)	0.468	0.057	44.1%	–
	HWE	9	[4,8,10,11,16,28,40–42]	1819/1710		0.885(0.764–1.025)	0.104	0.908(0.703–1.171)	0.456	0.029	53.3%	–
Caucasians	Total/HWE	5	[15,22,36,37,39]	1188/1102		**1.268(1.052–1.528)**	**0.013**	1.264(0.991–1.614)	0.060	0.163	38.8%	–
Tunisians	Total	2	[24,38]	510/599		0.860(0.676–1.094)	0.220	1.118(0.404–3.092)	0.830	0.001	91.7%	–
Han Chinese	Total	5	[11,23,40–42]	637/752		1.111(0.852–1.449)	0.436	1.121(0.664–1.891)	0.669	0.006	72.0%	–
Overall					Additive model (4a4a + 4b4b versus 4a4b)							
	Total	19	[4,8,10,11,15,16,18,19,22–24,28,36–42]	3859/3979		1.047(0.946–1.159)	0.371	1.008(0.845–1.202)	0.932	0.000	59.9%	0.440
	HWE	16	[4,8,10,11,15,16,18,22,28,36–42]	3458/3602		1.088(0.977–1.210)	0.124	1.062(0.892–1.265)	0.501	0.005	54.2%	0.713
Ethnicity												
Asians	Total	11	[4,8,10,11,16,19,23,28,40–42]	2093/1977		1.097(0.954–1.261)	0.195	1.073(0.892–1.291)	0.453	0.199	25.7%	–
	HWE	9	[4,8,10,11,16,28,40–42]	1819/1710		1.105(0.952–1.283)	0.189	1.076(0.856–1.352)	0.529	0.103	39.7%	–
Caucasians	Total/HWE	5	[15,22,36,37,39]	1188/1102		0.865(0.712–1.050)	0.143	0.865(0.703–1.064)	0.170	0.345	10.8%	–
Tunisians	Total	2	[24,38]	510/599		1.046(0.814–1.344)	0.724	0.738(0.213–2.562)	0.633	0.000	93.3%	–
Han Chinese	Total	5	[11,23,40–42]	637/752		0.934(0.710–1.231)	0.629	0.939(0.570–1.547)	0.805	0.018	66.6%	–
Overall					Homozygote model (4a4a versus 4b4b)							
	Total	19	[4,8,10,11,15,16,18,19,22–24,28,36–42]	3859/3979		0.974(0.793–1.197)	0.804	1.021(0.743–1.403)	0.897	0.064	35.4%	0.828
	HWE	16	[4,8,10,11,15,16,18,22,28,36–42]	3458/3602		0.946(0.759–1.179)	0.620	1.000(0.702–1.424)	1.000	0.068	37.1%	0.932
Ethnicity												
Asians	Total	11	[4,8,10,11,16,19,23,28,40–42]	2093/1977		0.889(0.663–1.193)	0.433	0.867(0.639–1.174)	0.356	0.456	0.0%	–
	HWE	9	[4,8,10,11,16,28,40–42]	1819/1710		0.839(0.615–1.145)	0.269	0.844(0.614–1.160)	0.297	0.689	0.0%	–
Caucasians	Total/HWE	5	[15,22,36,37,39]	1188/1102		**1.833(1.176–2.856)**	**0.007**	1.827(1.127–2.961)	0.014	0.331	13.1%	–
Tunisians	Total	2	[24,38]	510/599		0.722(0.453–1.150)	0.170	0.742(0.408–1.352)	0.330	0.209	36.8%	–
Han Chinese	Total	5	[11,23,40–42]	637/752		1.420(0.646–3.120)	0.382	1.460(0.634–3.366)	0.374	0.611	0.0%	–
Overall					Heterozygote model (4a4b versus 4b4b)							
	Total	19	[4,8,10,11,15,16,18,19,22–24,28,36–42]	3859/3979		0.952(0.858–1.055)	0.347	0.990(0.820–1.196)	0.920	0.000	64.3%	0.464
	HWE	16	[4,8,10,11,15,16,18,22,28,36–42]	3458/3602		0.915(0.821–1.021)	0.112	0.938(0.773–1.137)	0.513	0.001	61.3%	0.735
Ethnicity												
Asians	Total	11	[4,8,10,11,16,19,23,28,40–42]	2093/1977		0.904(0.784–1.043)	0.165	0.928(0.763–1.129)	0.457	0.146	31.6%	–
	HWE	9	[4,8,10,11,16,28,40–42]	1819/1710		0.892(0.765–1.039)	0.142	0.919(0.723–1.169)	0.493	0.073	44.3%	–
Caucasians	Total/HWE	5	[15,22,36,37,39]	1188/1102		1.198(0.985–1.458)	0.070	1.199(0.960–1.497)	0.110	0.288	20.0%	–
Tunisians	Total	2	[24,38]	510/599		0.912(0.707–1.178)	0.482	1.328(0.362–4.875)	0.669	0.000	93.4%	–
Han Chinese	Total	5	[11,23,40–42]	637/752		1.079(0.819–1.421)	0.590	1.075(0.645–1.793)	0.780	0.014	68.1%	–

The boldface: The *p* value was less than 0.05 and a significant association was found between eNOS (4a/4b) and the risk of incident DR. *N*: number of studies; OR: odd ratio; 95%CI: 95% confidence intervals; HWE: Hardy–Weinberg equilibrium.

**Table 4.** Result of source of control subgroup analysis of eNOS (4b/4a) polymorphism and DR risk in T2DM patients.

Variables	*N*	References	Case/Control	Genetic model	Fixed-effects model		Random-effects model		Heterogeneity	
					OR (95%CI)	*p* value	OR (95%CI)	*p* value	P_H_	I^2^ (%)
Source of control				Allelic model						
HB	10	[8,15,22–24,36,38–40,42]	1720/1990		1.066(0.945–1.201)	0.297	1.086(0.824–1.431)	0.558	0.000	77.9%
PB	9	[4,10,11,16,18,19,28,37,41]	2139/1989		0.901(0.806–1.006)	0.064	0.905(0.778–1.053)	0.197	0.200	27.4%
				Recessive model						
HB	10	[8,15,22–24,36,38–40,42]	1720/1990		0.961(0.714–1.294)	0.796	0.941(0.593–1.492)	0.794	0.060	45.0%
PB	9	[4,10,11,16,18,19,28,37,41]	2139/1989		1.014(0.772–1.331)	0.922	1.034(0.783–1.366)	0.813	0.613	0.0%
				Dominant model						
HB	10	[8,15,22–24,36,38–40,42]	1720/1990		1.084(0.940–1.249)	0.267	1.131(0.825–1.549)	0.445	0.000	76.3%
PB	9	[4,10,11,16,18,19,28,37,41]	2139/1989		**0.858(0.749–0.983)**	**0.027**	0.859(0.711–1.039)	0.118	0.136	35.2%
				Additive model						
HB	10	[8,15,22–24,36,38–40,42]	1720/1990		0.925(0.797–1.073)	0.301	0.863(0.639–1.164)	0.334	0.000	70.8%
PB	9	[4,10,11,16,18,19,28,37,41]	2139/1989		**1.168(1.016–1.341)**	**0.029**	1.162(0.985–1.371)	0.075	0.298	16.2%
				Homozygote model						
HB	10	[8,15,22–24,36,38–40,42]	1720/1990		1.097(0.812–1.482)	0.547	1.120(0.677–1.854)	0.659	0.027	52.1%
PB	9	[4,10,11,16,18,19,28,37,41]	2139/1989		0.879(0.663–1.164)	0.367	0.860(0.644–1.148)	0.307	0.464	0.0%
				Heterozygote model						
HB	10	[8,15,22–24,36,38–40,42]	1720/1990		1.084(0.933–1.260)	0.293	1.164(0.848–1.599)	0.348	0.000	73.4%
PB	9	[4,10,11,16,18,19,28,37,41]	2139/1989		**0.847(0.735–0.977)**	**0.022**	0.850(0.702–1.028)	0.094	0.172	30.8%

The boldface: The *p* value was less than 0.05 and a significant association was found between eNOS (4a/4b) and the risk of incident DR. PB: population-based; HB: hospital-based; *N*: number of studies; OR: odd ratio; 95%CI: 95% confidence intervals.

